# Structure and function of the telomeric CST complex

**DOI:** 10.1016/j.csbj.2016.04.002

**Published:** 2016-04-14

**Authors:** Cory Rice, Emmanuel Skordalakes

**Affiliations:** The Wistar Institute, and the Department of Biochemistry and Biophysics, University of Pennsylvania, Philadelphia PA 19104, USA

## Abstract

Telomeres comprise the ends of eukaryotic chromosomes and are essential for cell proliferation and genome maintenance. Telomeres are replicated by telomerase, a ribonucleoprotein (RNP) reverse transcriptase, and are maintained primarily by nucleoprotein complexes such as shelterin (TRF1, TRF2, TIN2, RAP1, POT1, TPP1) and CST (Cdc13/Ctc1, Stn1, Ten1). The focus of this review is on the CST complex and its role in telomere maintenance. Although initially thought to be unique to yeast, it is now evident that the CST complex is present in a diverse range of organisms where it contributes to genome maintenance. The CST accomplishes these tasks via telomere capping and by regulating telomerase and DNA polymerase alpha-primase (polα-primase) access to telomeres, a process closely coordinated with the shelterin complex in most organisms. The goal of this review is to provide a brief but comprehensive account of the diverse, and in some cases organism-dependent, functions of the CST complex and how it contributes to telomere maintenance and cell proliferation.

## 1 Introduction

Telomeres compose the non-coding ends of eukaryotic chromosomes and play a crucial role in the protection and replication of our genome [1], [2]. Eukaryotic chromosomes, unlike prokaryotes are linear, presenting the cell with a unique problem: telomere ends can be recognized as DNA strand breaks by the recombination and repair systems of the cell, which would lead to chromosome end-to-end fusion and genomic instability or apoptosis [3], [4]. Telomeres together with telomere binding complexes, such as shelterin, repress unwanted DNA damage response (DDR) and serve as a buffer between essential genomic information and the ends of chromosomes. They also promote the full replication of our genome, thus preventing senescence, which is usually associated with significant telomere shortening [5], [6].

**Fig. 1:**
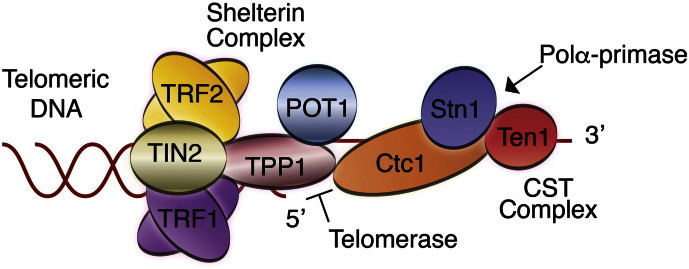
Schematic of the sheleterin and CST complexes bound to telomeric DNA. The role of TPP1 in recruiting telomerase to telomeres and its regulation by the CST complex are highlighted.

Proper telomere length regulation and maintenance are essential for genome stability. There are at least two complexes that contribute to telomere maintenance: shelterin [7], [8] and CST [9], [10]. Shelterin is a six subunit complex consisting of TRF1, TRF2, TIN2, RAP1, POT1, TPP1, and localizes specifically to double- and single-stranded telomeric DNA (Fig. 1) [11]. Although there is still a lot to learn about the role of shelterin at telomeres, work from a confluence of labs has shown that it is critical for suppressing DDR at telomeres, thus preventing chromosome fusions [11]. Shelterin also caps the ends of chromosomes by facilitating T-loop formation and by sequestering the single-stranded DNA portion of the telomere [12], [13]. It also acts as a telomerase processivity factor by recruiting telomerase to telomeres [14], [15].

The CST is a trimeric complex composed of Ctc1, Stn1, and Ten1 in higher eukaryotes and Cdc13, Stn1, and Ten1 in yeast (*Saccharomyces cerevisiae*) [9], [16]. CST localizes specifically to the single-stranded telomeric DNA, including the telomeric overhang where it is involved in chromosome end capping and telomere length regulation (Fig. 1) [9], [17], [18], [19], [20]. However, there is increasing evidence, which suggests that the Stn1-Ten1, CST sub-complex has extra-telomeric functions. Current data show that Stn1-Ten1 act as a replication protein-A, complex (RPA)-like complex, rescuing genome-wide replication fork stalling during conditions of replication stress [16], [21], [22]. The RPA, is a heterotrimeric protein complex that binds non-specifically single-stranded DNA and is involved in a wide array of DNA metabolic pathways including DNA replication and DNA damage cellular responses [23]. It is worth noting that in vertebrates the capping properties of the vertebrate CST may be dispensable in vivo due to the presence of shelterin, which also caps the ends of chromosomes [11].

Proper telomere maintenance is critical to genome stability. Mutations in genes that encode essential telomere components result in some of the most intractable diseases. Human telomere dysfunction is known to cause symptoms of pre-mature aging, pulmonary fibrosis, and bone marrow failure as well as an increased incidence of cancer [24], [25], [26], [27], [28], [29], [30]. Further insight in the molecular mechanisms of telomere maintenance will allow us to better understand the role of telomere dysfunction in human disease.

## 2 Telomere replication

The linear nature of eukaryotic chromosomes results in telomere shortening over time [31]. Known as the “end-replication problem”, synthesis of the lagging-strand requires RNA priming for the replication of the Okazaki fragments, the DNA fragments complementary to the lagging strand of the chromosome [32]. The requirement of RNA primers for the full replication of the lagging strand prevents its full replication, leading to loss of 50–200 bases of telomeric DNA with every cell division [33]. When telomeres become critically short, the cell enters a non-replicative state known as cellular senescence followed by apoptosis [34], [35].

To overcome the end-replication problem, a specialized enzyme is recruited to the ends of chromosomes to help replicate telomeres. Telomeres are replicated during the S-phase (late S-phase in yeast) by telomerase, a ribonucleoprotein reverse transcriptase [36], [37], [38], [39], [40], [41], [42]. Unlike most polymerases, telomerase consists of a protein subunit (TERT) and an integral RNA component (TER), which TERT uses to add multiple, identical repeats of DNA (telomeres) to the ends of chromosomes [37].

G-strand (telomeric sense-strand) synthesis by telomerase is followed by replication of the C-strand (telomeric antisense-strand) by polα-primase during late S early G2 phase [43]. Limited evidence, primarily from work carried out in *Euplotes crassus* and HeLa cells, suggests that the switch between G- to C-strand synthesis is a highly coordinated event generating a homogeneous C- and heterogeneous G-strand telomeres in *Euplotes*[33], [42], [44], [45]. Although the precise mechanism for this switch is not clear, current evidence suggest that is mediated by DNA polα [44].

## 3 Telomere structure

Even though telomeric DNA comprises the non-coding portion of the chromosome, it is merely a passive structure. Telomeric DNA adopts at least two well-defined tertiary structures, the T-loop and the G-quadruplexes (G-quads), both of which serve to regulate telomere length and protect the ends of chromosomes [12], [46], [47]. The telomeric ends of eukaryotic chromosomes are composed of repetitive, G-rich, non-coding, DNA repeats (TTAGGG in mammals). The G-rich nature of telomeric DNA promotes the formation of higher-order structures, known as G-quads [47]. G-quads are formed when four or more guanine bases come together through Hoogsteen hydrogen bonds to form a planar structure, frequently stabilized by cations like potassium [48]. The formation of telomeric G-quads has been shown both in vitro and in vivo, and is known to interfere with the elongation of telomeres and most likely hinder exonuclease degradation. It is therefore possible that G-quad formation is a regulatory mechanism of the telomere elongation and protection pathway [49], [50], [51], [52]. The presence of stable G-quads throughout the single-stranded, telomeric DNA can pose challenges for the telomere replication machinery. For telomeres to be replicated and maintained, the G-quads must be resolved. Several eukaryotic proteins have been reported to resolve G- quads, including the *S. cerevisiae* Cdc13 protein, the *C. glabrata* CST complex [53], [54], POT1 [55], [56], [57] and the RTEL1, and RecQ Werner's and Bloom's syndrome helicases [58], [59], [60], [61].

T-loops on the other hand are generated when the single-stranded G-overhang invades the duplex DNA to form a loop-like structure [12]. T-loop formation is promoted and stabilized by the components of the shelterin complex such as TRF2 and RAP1 [13], [62], [63]. Like G-quads, T-loops provide a regulatory mechanism of telomere elongation and protection. T-loops were also reported recently to form compact nucleoprotein structures, thus acting like nucleosomes specific to telomeric regions of the chromosome [64], [65].

## 4 Conservation of the CST complex

Until recently the CST complex was thought to be unique to yeast, however, recent findings indicate that the CST complex may be universally conserved [66], [67], [68]. Despite the presence of the CST complex in ciliates, yeast, plants, and mammals, low or complete lack of sequence identity and emerging differences across species, raises significant questions regarding the functional conservation of this complex. For example, the yeast and human CST components, Stn1 and Ten1, are highly conserved structurally [69]. However, the major components of the CST complex (Cdc13 and Ctc1) have no sequence identity and vary significantly in length and to some extent in function. For example, yeast Cdc13 is known to recruit telomerase to telomeres via its interaction with Est1, a component of the yeast telomerase holoenzyme [70], [71]. In contrast, human Ctc1 is known to directly inhibit telomerase recruitment to telomeres [18]. What makes things even more complex is the recent identification of the CST complex in ciliates. The p75-p45-p19 of *Tetrahymena thermophila*, which has no sequence identity to any known CST complexes, has been proposed to act as the ciliate CST complex and to coordinate G- and C-strand synthesis [68].

## 5 Cdc13/Ctc1 structure function

**Fig. 2:**
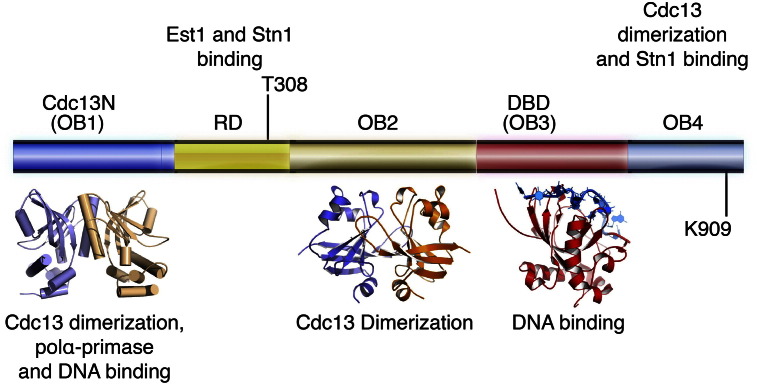
Primary and tertiary structure of *S. cerevisiae* Cdc13. Primary structure of Cdc13 indicating domain organization. Atomic structures of each of the yeast Cdc13 domains are also shown ((OB1 (PDB ID:3NWS), OB2 (PDB ID:4HCE), DBD (PDB ID:1KXL)). Key post-translational modifications known to contribute to Cdc13 function are also indicated.

Structural studies of CST components have revealed that recognition of the single-stranded telomeric overhangs is mediated by several oligosaccharide/oligonucleotide-binding folds (OB-folds) present in all three subunits of the CST complex [72]. OB-folds are usually a five-stranded, closed, beta barrel motif, known to bind single-stranded nucleic acid and polypeptides [73], [74]. The main component of the yeast CST, Cdc13, consists of four OB-folds [20], [75], [76], [77], [78], [79], [80]. Subtle but distinct differences between these four OB-folds, allows Cdc13 engagement in a wide range of processes including single-stranded DNA binding, Cdc13 homo-dimerization, and polα-primase binding (Fig. 2) [76], [77], [81].

The Cdc13 N-terminal domain comprises an OB-fold (OB1 - PDB ID:3NWS and 3OIP) that is involved in a wide range of functions related to telomere length regulation. It assists in Cdc13 homo-dimerization, a process we postulated to be important for telomerase loading to telomeres [77], [81]. Cdc13 also recruits telomerase to telomeres, a process mediated by the telomerase associated protein Est1 [75]. Since telomerase is thought to act both as a monomer and a dimer [82], [83], the dimeric state of Cdc13 may assist the dimeric form of telomerase for telomere loading and synthesis. The Cdc13 (OB1) has also been shown to bind and recruit polα to telomeres [84] most likely a coordinated effort with Stn1 [85], [86], [87], [88], [89]. Moreover Cdc13 (OB1) has weak, non-specific, single-stranded DNA binding activity [81]. We currently hypothesize that this additional property of Cdc13 (OB1) may be important a) for regulating telomerase access to telomeres during the various steps of the cell cycle, and b) for the proper loading of polα-primase to telomeres for C-strand synthesis.

The Cdc13 recruitment domain (Cdc13RD) is an unstructured region of the protein (Fig. 2) containing a large number of phosphorylation sites implicated in two distinct CST functions [90], [91], [92], [93], [94], [95]. Phosphorylation of the Cdc13RD promotes Est1 binding and telomerase recruitment to telomeres [90], [92]. De-phosphorylation of Cdc13RD promotes CST (Cdc13-Stn1-Ten1) assembly via association of Stn1 with the Cdc13RD, a state of the complex known to bind and cap the ends of chromosomes [70], [92], [93], [94], [95].

Following the Cdc13RD is the Cdc13 (OB2). Interestingly, the Cdc13 (OB2) domain contains unusually long, surface loops also involved in Cdc13 (OB2) homo-dimerization (PDB ID:4HCE). Unlike Cdc13 (OB1), the OB2 is not directly involved in protein or nucleic acid binding. Instead Cdc13 (OB2) dimerization promotes the faithful binding of Stn1 to the Cdc13RD domain and the proper assembly of the CST complex and telomere capping [76].

Immediately following the Cdc13 (OB2) is a third OB-fold referred to as the DNA Binding Domain (DBD - PDB ID:1KXL) of Cdc13 due to its high affinity and specificity for approximately 11 bases of single-stranded telomeric DNA [96]. Binding of Cdc13 (DBD) to telomeric DNA assists in the localization of the yeast Cdc13 to the telomeric overhang.

Although there is no structure of the *S. cerevisiae* C-terminal domain of Cdc13, structures from *Candida glabrata* show that it is an OB-fold (OB4 - PDB ID:3RMH) also involved in Cdc13 dimerization and is also proposed to enhance Cdc13 single-stranded, telomeric DNA binding [80].

Like Cdc13, Ctc1 is predicted to consist of multiple OB-folds [16], [67], [74]. Due to lack of sequence identity between Cdc13 and Ctc1, it has been difficult to accurately predict the domain organization of Ctc1 and no structural information currently exists.

## 6 Stn1-Ten1 structure function

Stn1 and Ten1, the most conserved components of the CST, form a stable complex in vitro [69], [71], [72], [73], [74], [75], [76], [77], [78], [79], [80], [81], [82], [83], [84], [85], [86], [87], [88]. Ten1 is the smallest of the three CST components and contains a single OB-fold with a highly conserved C-terminal helix (4JOI, 3KF6, 3KF8, 3K0X). The yeast Ten1 has been shown to bind single-stranded, telomeric DNA with weak affinity, an interaction that is proposed to enhance the DNA binding activity of Cdc13 [100]. Unlike Ten1, Stn1 consists of an N-terminal OB-fold and two wing-helix-turn-helix (wHTH) motifs (PDB ID:4JOI, 3KF6, 3KF8, 3K10, 3KEY, 4JQF). The Stn1 OB-fold, like Ten1, contains a highly conserved C-terminal helix. The Stn1-Ten1 complex comes together via extensive interactions between the two C-terminal helices of the OB-folds as well as contacts between the bodies of these domains. Stn1-Ten1 association positions the putative DNA binding pockets of the two proteins in parallel with each other thus forming an extensive nucleic acid binding pocket on the surface of the complex [69]. The wHTH motifs have been implicated in polα and Cdc13 binding [69], [85], [86], [88].

**Fig. 3:**
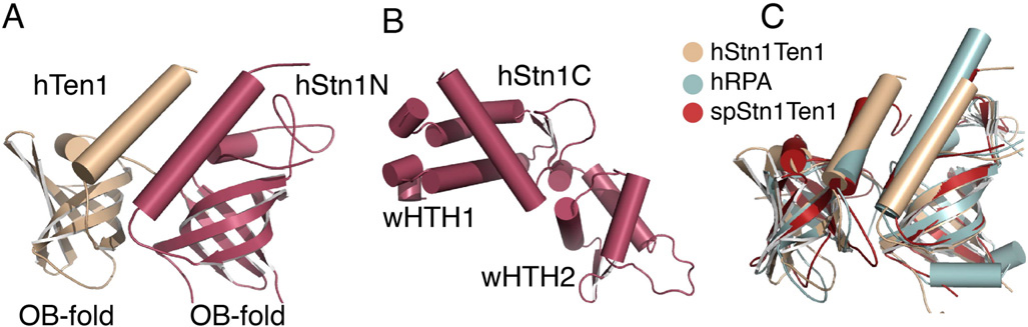
X-ray, crystal structure of the Stn1-Ten1 complex. A) Structure of the human Stn1-Ten1 (hStn1-Ten1) complex (PDB ID:4JOI); only the N-terminal OB fold of Stn1 (hStn1N) is involved in contacts with Ten1. B) Structure of the C-terminal domain of human Stn1 (hStn1C – PDB ID: 4JQF) consisting of two wHTH motifs. C) Overlay of the human, *S. pombe* Stn1-Ten1 (PDB ID:3KF6) and human RPA (PDB ID:1QUQ) complexes. The overlay shows a striking similarity between the three complexes.

Structural data on the human CST is currently limited to Stn1-Ten1, which is similar to the yeast complex (Fig. 3A and B) [69]. Interestingly, unlike the yeast Ten1, the human homolog does not bind single-stranded nucleic acid [69]. This is not surprising if one takes into consideration the lack of residue conservation in the putative DNA binding pocket of Ten1 [69]. In contrast to Ten1, human Stn1 binds single-stranded DNA with 2 μM binding affinity and no specificity [69]. High affinity and specificity of the human CST complex for single-stranded telomeric DNA is provided by the larger component of CST, Ctc1 [18].

All evidence so far points to tight and specific association of Cdc13/Ctc1 with single-stranded telomeric DNA, which assists in the localization of the CST complex to telomeres [18], [19], [20]. Cdc13, Ctc1, and Stn1 are also involved in a series of protein–protein interactions, which contribute to telomerase and polα-primase recruitment to telomeres for G- and C-strand synthesis respectively. What remains a mystery is the precise role of human Ten1 in telomere biology. Ten1 is essential for proper telomere capping, however, the absence of any evidence for nucleic acid or protein binding raises questions regarding its role in telomere biology. Currently, Ten1 has been shown to enhance the telomeric DNA-binding activity of Cdc13, although Ten1 itself exhibits weak DNA binding activity in yeast and no affinity in humans [69], [98]. Another possibility is that it acts as a steric block preventing access of the telomeric overhang bound by Cdc13/Ctc1 and Stn1 for telomere elongation, which would be consistent with the telomere uncapping defects associated with a dysfunctional CST complex [99].

## 7 Stn1-Ten1 is an RPA like complex

Interestingly, Stn1-Ten1 domain composition and organization is strikingly similar to that of the small subunits (RPA32 and RPA14) of the Replication Protein A (RPA) complex (Fig. 3C) [66], [67], [97]. The most striking difference between Stn1-Ten1 and RPA is the presence of two wHTH motifs in Stn1, whereas RPA32 contains only one [97]. Despite the overall structural conservation of the Stn1-Ten1 and RPA14–32 complexes, current evidence suggests that CST and RPA similarities are limited to these two subunits of the two complexes [80].

wHTH motifs are known for protein–protein interactions or double-stranded nucleic acid binding. There is no evidence that the two wHTH motifs of Stn1 are involved in DNA binding; in fact, the organization of the two Stn1 wHTH motifs occludes their putative, double stranded, nucleic acid binding pocket. Instead, like the RPA32 wHTH motif, the C-terminal domain of Stn1 is thought to interact with Cdc13 during CST complex formation and telomere capping [100], [101]. It is also involved in the recruitment of polα-primase thus facilitating C-strand synthesis at telomeres [89], [102].

## 8 The CST complex and telomere replication

Telomere elongation is a tightly regulated process as it maintains the proliferative nature of the cell and yet prevents cellular immortalization associated with carcinogenesis [70]. The yeast CST complex is known to both downregulate and upregulate telomere elongation [71], [90], [92], [93], [103]. Yeast CST downregulates telomere elongation through tight and specific interaction with the telomeric overhang, a process known as telomere capping [70]. Binding of CST to the end of chromosomes sequesters the telomeric overhang thus preventing access of telomerase to the 3′-end of the DNA for telomere replication [20]. It is important to emphasize that all three components of the yeast CST (Cdc13, Stn1 and Ten1) are required for telomere capping. Loss of any of the yeast CST subunits or limited disruption of the CST assembly leads to telomere uncapping and telomere length elongation. Telomere elongation suggests that the telomeric overhang has become accessible to telomerase [69], [76], [77], [81].

Interestingly, the yeast CST complex also contributes to the upregulation of telomere length via recruitment of telomerase to telomeres [70], [75], [78], [79]. As we mentioned earlier, telomerase recruitment to yeast telomeres is mediated by the Cdc13RD and its interaction with the telomerase associated protein, Est1. Est1 is a predicted 14-3-3 protein fold that has affinity for phosphorylated peptides [104].

The switch between the capping and elongation state of the CST is heavily influenced by phosphorylation of Cdc13RD and SUMOylation of the Cdc13 (OB4) (Fig. 2). In fact, the Zakian and Garcia labs recently identified 21 in vivo, Cdc13 phosphorylation sites [95]. Although the function of most of these sites is currently unknown they all are responsible for the tight regulation of the CST complex and therefore of telomere length.

During late S to early G2 phase, Cdc13RD phosphorylation of Cdc13RD residue T308 by the kinase Cdk1 allows for Est1 binding to the Cdc13RD and recruitment of telomerase to telomeres [90], [92], [105]. Est1 binding to Cdc13 is further facilitated by the cell cycle-dependent SUMOylation of Cdc13 (OB4) residue Lys909 [101], [106]. Cdc13RD phosphorylation and Cdc13 (OB4) SUMOylation partially disrupt Cdc13-Stn1 binding and therefore the productive, capping state of the CST complex. Disruption of the CST complex allows for release of the telomeric overhang and Est1-dependent telomerase recruitment to telomeres for G-strand elongation.

The CST complex also promotes telomere, C-strand synthesis via recruitment of polα to telomeres [17], [70], [86], [87], [102], [107]. Current evidence suggests that polα-primase recruitment to telomeres is mediated by Cdc13/Ctc1 and Stn1 [16], [70], [86], [102], [108]. Cdc13-dependent polα-primase recruitment to telomeres involves at least the N-terminal domain of Cdc13 (OB1). Earlier studies identified two distinct regions of polα-primase making direct contacts with Cdc13 (OB1) and include residues 13–392 and 47–560 [78], [109], while more recent structural data suggests a helix consisting of residues 215–250 binds the Cdc13 (OB1) [84].

A striking difference between the yeast and human CST complexes lies with the mechanism of telomerase recruitment to telomeres. We have so far stated that the yeast CST, and in particular Cdc13, recruit telomerase to the ends of chromosomes for telomere elongation. In higher eukaryotes and in particular humans, the Pot1-TPP1 sub-complex of shelterin mediates this process. The Cech lab and others have shown that telomerase binds directly to the N-terminal OB-fold of TPP1 (TEL patch), an interaction that assists in bringing telomerase to telomeres and enhances its processivity [15], [110], [111]. Interestingly, the Lingner lab has shown that the human CST complex downregulates telomerase recruitment to telomeres to one cycle of telomere replication per cell cycle via direct contact with TPP1 [18]. It is worth noting that the yeast telomerase associated protein Est3 is structurally similar to TPP1 and has been proposed to share similar functional roles in telomeres [112]. The fact that TPP1 is conserved in humans and possibly yeast, suggests that the CST may have dual roles in telomerase recruitment and inhibition to telomeres adding an additional regulatory step in the repertoire of CST functions.

## 9 Extra-telomeric functions of CST

Recent studies have shown that the human CST complex may have additional functions beyond the telomeres. Work form the Price lab has shown that the CST complex rescues genome-wide (telomeric and non-telomeric) replication fork stalling during conditions of replication stress by facilitating dormant origin firing [21], [113]. Although the CST has been thought to work exclusively at telomeres, the human Stn1-Ten1 complex binds single-stranded DNA weakly, in a non-specific manner [69]. In addition, Stn1-Ten1 is an RPA-like complex, which is known to participate in genome-wide replication. Taken together the data indicates that Stn1 and Ten1 may have a dual role in stabilizing single-stranded DNA and assisting in DNA replication throughout the genome and at telomeres.

## 10 CST and human disease

Naturally occurring mutations in telomeric complexes are associated with aplastic anemia, pulmonary fibrosis, Coats plus (CP) and Dyskeratosis Congenita (DC) [29], [114]. Although there is no current evidence implicating Stn1 or Ten1 in human disease, there are a number of naturally occurring Ctc1 mutations, which result in a range of rare genetic disorders such as CP and DC. CP is characterized by intracranial calcifications, hematological abnormalities, and retinal vascular defects [25], [26], [27], while DC is an inherited bone marrow failure syndrome [28], [30]. Several patients with CP display critically shortened telomeres, suggesting that telomerase dysfunction plays an important role in disease pathogenesis. Moreover, a wealth of recent data suggests a direct correlation between cardiovascular disease or infectious disease, and shorter telomeres in blood cells [115]. Defects in telomere structure and protection, independent of length, were also reported in Hoyeraal–Hreidarsson syndrome [116].

**Fig. 4:**
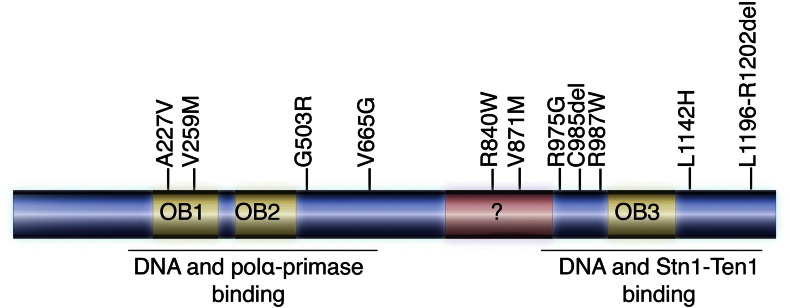
Primary structure of human Ctc1. Predicted domains and naturally occurring mutations associated with human disease are indicated.

Ctc1 mutations associated with human disease are typically biallelic, and in some cases severe frame shift mutations that lead to a truncated Ctc1 and complete loss of function [26], [27]. Work from the Lingner lab has shown that these mutations act by disrupting CST complex formation, telomeric DNA binding, polα-primase recruitment to telomeres, and/or cellular localization of the complex in vivo (Fig. 4). It is worth noting that the Chang lab also carried out this study in mice using murine Ctc1 and did not observe the same phenotypes as the Lingner lab. It is therefore possible that the Ctc1-mutant associated defects observed could be organism-dependent [27]. Current models of Ctc1 suggest that the N-terminus of the protein consists of two OB folds [74]. Three naturally occurring mutations within the N-terminal and central region of Ctc1 (A227V, V259M, and V665G) disrupt Ctc1/polα-primase binding (Fig. 4). In particular, the V259M mutation resulted in significant accumulation of telomere-free ends, while the G503R one resulted in elongated telomeres, a defect usually associated with a dysfunctional CST complex [26]. The Ctc1 disease mutations L1142H and 1196-Δ7 (deletion of amino acid residues 1196–1202) disrupts Ctc1-Stn1 association, and polα-primase recruitment to telomeres (Fig. 4). In addition to disrupting CST assembly and telomere maintenance, the A227V, V259M, R987, L1142H mutations and 1196-Δ7 deletion also negatively impacted the nuclear localization of these proteins. The role of the Ctc1, naturally occurring mutations R840W and V871M in human disease is currently unclear. Interestingly, none of the identified Ctc1 mutations interfere with the Pot1-TPP1 inhibitory properties of CST.

## 11 Summary and outlook

Telomeres allow for the full replication of our genome and prevent deleterious events such as chromosome fusions, and exonucleolytic degradation. Dysfunctional telomeres can lead to genomic instability, the hallmark of cancer or cell cycle arrest, senescence and apoptosis. Telomeres accomplish this task together with specialized proteins, such as Cdc13/Ctc1, Stn1 and Ten1, which together assemble into what is commonly known as the CST [117], an RPA-like complex [66], [67], [97].

Telomere length regulation by the trimeric CST complex is key to genome maintenance. Current evidence shows that both the human and yeast CST complexes localize to telomeres through association with the telomeric overhang. They also regulate access of telomerase and polα-primase to the end of chromosomes for G- and C-strand synthesis, respectively.

Naturally occurring mutations of these nucleoprotein complexes are associated with aplastic anemia, pulmonary fibrosis and a range of rare genetic disorders such as CP and DC. There is also data, which suggests a direct link between cardiovascular or infectious disease and shorter telomeres in blood cells, while defects in telomere structure and protection have been reported in Hoyeraal–Hreidarsson syndrome. Understanding the mechanisms that regulate and maintain the integrity of telomeres is paramount to identifying therapies for the treatment of some of the most intractable diseases such as cancer.

Currently, the biophysical mechanisms underlying CST architecture and function are poorly understood. Structural, biochemical and functional characterization of these factors, both in isolation and in complex with one another, is needed to answer a number of questions regarding the role of this complex in telomere biology and genome integrity.

## References

[1] Blackburn E.H., Gall J.G. (1978). A tandemly repeated sequence at the termini of the extrachromosomal ribosomal RNA genes in Tetrahymena. J Mol Biol.

[2] de Lange T. (2009). How telomeres solve the end-protection problem. Science.

[3] Longhese M.P. (2008). DNA damage response at functional and dysfunctional telomeres. Genes Dev.

[4] Maser R.S., DePinho R.A. (2004). Telomeres and the DNA damage response: why the fox is guarding the henhouse. DNA Repair (Amst).

[5] Effros R.B., Walford R.L. (1984). T cell cultures and the Hayflick limit. Hum Immunol.

[6] Greider C.W., Blackburn E.H. (1996). Telomeres, telomerase and cancer. Sci Am.

[7] Palm W., de Lange T. (2008). How shelterin protects mammalian telomeres. Annu Rev Genet.

[8] Baumann P., Podell E., Cech T.R. (2002). Human Pot1 (protection of telomeres) protein: cytolocalization, gene structure, and alternative splicing. Mol Cell Biol.

[9] Wellinger R.J. (2009). The CST complex and telomere maintenance: the exception becomes the rule. Mol Cell.

[10] Giraud-Panis M.J., Teixeira M.T., Geli V., Gilson E. (2010). CST meets shelterin to keep telomeres in check. Mol Cell.

[11] de Lange T. (2005). Shelterin: the protein complex that shapes and safeguards human telomeres. Genes Dev.

[12] Doksani Y., Wu J.Y., de Lange T., Zhuang X. (2013). Super-resolution fluorescence imaging of telomeres reveals TRF2-dependent T-loop formation. Cell.

[13] Griffith J.D., Comeau L., Rosenfield S., Stansel R.M., Bianchi A., Moss H. (1999). Mammalian telomeres end in a large duplex loop. Cell.

[14] Wang F., Lei M. (2011). Human telomere POT1-TPP1 complex and its role in telomerase activity regulation. Methods Mol Biol.

[15] Wang F., Podell E.R., Zaug A.J., Yang Y., Baciu P., Cech T.R. (2007). The POT1-TPP1 telomere complex is a telomerase processivity factor. Nature.

[16] Price C.M., Boltz K.A., Chaiken M.F., Stewart J.A., Beilstein M.A., Shippen D.E. (2010). Evolution of CST function in telomere maintenance. Cell Cycle.

[17] Nakaoka H., Nishiyama A., Saito M., Ishikawa F. (2012). *Xenopus laevis* Ctc1-Stn1-Ten1 (xCST) protein complex is involved in priming DNA synthesis on single-stranded DNA template in Xenopus egg extract. J Biol Chem.

[18] Chen L.Y., Redon S., Lingner J. (2012 Aug 23). The human CST complex is a terminator of telomerase activity. Nature..

[19] Hughes T.R., Weilbaecher R.G., Walterscheid M., Lundblad V. (2000). Identification of the single-strand telomeric DNA binding domain of the *Saccharomyces cerevisiae* Cdc13 protein. Proc Natl Acad Sci U S A.

[20] Lin J.J., Zakian V.A. (1996). The Saccharomyces CDC13 protein is a single-strand TG1-3 telomeric DNA-binding protein in vitro that affects telomere behavior in vivo. Proc Natl Acad Sci U S A.

[21] Stewart J.A., Wang F., Chaiken M.F., Kasbek C., Chastain P.D., Wright W.E. (2012). Human CST promotes telomere duplex replication and general replication restart after fork stalling. EMBO J.

[22] Wang F., Stewart J., Price C.M. (2014). Human CST abundance determines recovery from diverse forms of DNA damage and replication stress. Cell Cycle.

[23] Wold M.S. (1997). Replication protein A: a heterotrimeric, single-stranded DNA-binding protein required for eukaryotic DNA metabolism. Annu Rev Biochem.

[24] Armanios M., Blackburn E.H. (2012). The telomere syndromes. Nat Rev Genet.

[25] Anderson B.H., Kasher P.R., Mayer J., Szynkiewicz M., Jenkinson E.M., Bhaskar S.S. (2012). Mutations in CTC1, encoding conserved telomere maintenance component 1, cause Coats plus. Nat Genet.

[26] Chen L.Y., Majerska J., Lingner J. (2013). Molecular basis of telomere syndrome caused by CTC1 mutations. Genes Dev.

[27] Gu P., Chang S. (2013). Functional characterization of human CTC1 mutations reveals novel mechanisms responsible for the pathogenesis of the telomere disease Coats plus. Aging Cell.

[28] Keller R.B., Gagne K.E., Usmani G.N., Asdourian G.K., Williams D.A., Hofmann I. (2012). CTC1 Mutations in a patient with dyskeratosis congenita. Pediatr Blood Cancer.

[29] Vulliamy T., Marrone A., Dokal I., Mason P.J. (2002). Association between aplastic anaemia and mutations in telomerase RNA. Lancet.

[30] Walne A.J., Bhagat T., Kirwan M., Gitiaux C., Desguerre I., Leonard N. (2013). Mutations in the telomere capping complex in bone marrow failure and related syndromes. Haematologica.

[31] Levy M.Z., Allsopp R.C., Futcher A.B., Greider C.W., Harley C.B. (1992). Telomere end-replication problem and cell aging. J Mol Biol.

[32] Okazaki R., Okazaki T., Sakabe K., Sugimoto K. (1967). Mechanism of DNA replication possible discontinuity of DNA chain growth. Jpn J Med Sci Biol.

[33] Zhao Y., Shay J.W., Wright W.E. (2011). Telomere terminal G/C strand synthesis: measuring telomerase action and C-rich fill-in. Methods Mol Biol.

[34] Allsopp R.C., Vaziri H., Patterson C., Goldstein S., Younglai E.V., Futcher A.B. (1992). Telomere length predicts replicative capacity of human fibroblasts. Proc Natl Acad Sci U S A.

[35] Counter C.M., Avilion A.A., LeFeuvre C.E., Stewart N.G., Greider C.W., Harley C.B. (1992). Telomere shortening associated with chromosome instability is arrested in immortal cells which express telomerase activity. EMBO J.

[36] Gillis A.J., Schuller A.P., Skordalakes E. (2008). Structure of the *Tribolium castaneum* telomerase catalytic subunit TERT. Nature.

[37] Greider C.W., Blackburn E.H. (1987). The telomere terminal transferase of Tetrahymena is a ribonucleoprotein enzyme with two kinds of primer specificity. Cell.

[38] Mitchell M., Gillis A., Futahashi M., Fujiwara H., Skordalakes E. (2010). Structural basis for telomerase catalytic subunit TERT binding to RNA template and telomeric DNA. Nat Struct Mol Biol.

[39] Tomlinson R.L., Ziegler T.D., Supakorndej T., Terns R.M., Terns M.P. (2006). Cell cycle-regulated trafficking of human telomerase to telomeres. Mol Biol Cell.

[40] Wellinger R.J., Wolf A.J., Zakian V.A. (1993). Saccharomyces telomeres acquire single-strand TG1-3 tails late in S phase. Cell.

[41] Ten Hagen K.G., Gilbert D.M., Willard H.F., Cohen S.N. (1990). Replication timing of DNA sequences associated with human centromeres and telomeres. Mol Cell Biol.

[42] Wright W.E., Tesmer V.M., Liao M.L., Shay J.W. (1999). Normal human telomeres are not late replicating. Exp Cell Res.

[43] Hubscher U., Maga G., Spadari S. (2002). Eukaryotic DNA polymerases. Annu Rev Biochem.

[44] Fan X., Price C.M. (1997). Coordinate regulation of G- and C strand length during new telomere synthesis. Mol Biol Cell.

[45] Zhao Y., Sfeir A.J., Zou Y., Buseman C.M., Chow T.T., Shay J.W. (2009). Telomere extension occurs at most chromosome ends and is uncoupled from fill-in in human cancer cells. Cell.

[46] Rhodes D., Giraldo R. (1995). Telomere structure and function. Curr Opin Struct Biol.

[47] Henderson E., Hardin C.C., Walk S.K., Tinoco I., Blackburn E.H. (1987). Telomeric DNA oligonucleotides form novel intramolecular structures containing guanine-guanine base pairs. Cell.

[48] Tran P.L., Mergny J.L., Alberti P. (2011). Stability of telomeric G-quadruplexes. Nucleic Acids Res.

[49] Biffi G., Tannahill D., McCafferty J., Balasubramanian S. (2013). Quantitative visualization of DNA G-quadruplex structures in human cells. Nat Chem.

[50] Schaffitzel C., Berger I., Postberg J., Hanes J., Lipps H.J., Pluckthun A. (2001). In vitro generated antibodies specific for telomeric guanine-quadruplex DNA react with Stylonychia lemnae macronuclei. Proc Natl Acad Sci U S A.

[51] Oganesian L., Moon I.K., Bryan T.M., Jarstfer M.B. (2006). Extension of G-quadruplex DNA by ciliate telomerase. EMBO J.

[52] Zahler A.M., Williamson J.R., Cech T.R., Prescott D.M. (1991). Inhibition of telomerase by G-quartet DNA structures. Nature.

[53] Lue N.F., Zhou R., Chico L., Mao N., Steinberg-Neifach O., Ha T. (2013). The telomere capping complex CST has an unusual stoichiometry, makes multipartite interaction with G-Tails, and unfolds higher-order G-tail structures. PLoS Genet.

[54] Lin Y.C., Shih J.W., Hsu C.L., Lin J.J. (2001). Binding and partial denaturing of G-quartet DNA by Cdc13p of *Saccharomyces cerevisiae*. J Biol Chem.

[55] Wang H., Nora G.J., Ghodke H., Opresko P.L. (2011). Single molecule studies of physiologically relevant telomeric tails reveal POT1 mechanism for promoting G-quadruplex unfolding. J Biol Chem.

[56] Zaug A.J., Podell E.R., Cech T.R. (2005). Human POT1 disrupts telomeric G-quadruplexes allowing telomerase extension in vitro. Proc Natl Acad Sci U S A.

[57] Colgin L.M., Baran K., Baumann P., Cech T.R., Reddel R.R. (2003). Human POT1 facilitates telomere elongation by telomerase. Curr Biol.

[58] Barefield C., Karlseder J. (2012). The BLM helicase contributes to telomere maintenance through processing of late-replicating intermediate structures. Nucleic Acids Res.

[59] Crabbe L., Verdun R.E., Haggblom C.I., Karlseder J. (2004). Defective telomere lagging strand synthesis in cells lacking WRN helicase activity. Science.

[60] Mohaghegh P., Karow J.K., Brosh R.M., Bohr V.A., Hickson I.D. (2001). The Bloom's and Werner's syndrome proteins are DNA structure-specific helicases. Nucleic Acids Res.

[61] Vannier J.B., Pavicic-Kaltenbrunner V., Petalcorin M.I., Ding H., Boulton S.J. (2012). RTEL1 dismantles T loops and counteracts telomeric G4-DNA to maintain telomere integrity. Cell.

[62] Stansel R.M., de Lange T., Griffith J.D. (2001). T-loop assembly in vitro involves binding of TRF2 near the 3′ telomeric overhang. EMBO J.

[63] Grunstein M. (1997). Molecular model for telomeric heterochromatin in yeast. Curr Opin Cell Biol.

[64] Bandaria J.N., Qin P., Berk V., Chu S., Yildiz A. (2016). Shelterin protects chromosome ends by compacting telomeric chromatin. Cell.

[65] de Lange T. (2004). T-loops and the origin of telomeres. Nat Rev Mol Cell Biol.

[66] Gao H., Cervantes R.B., Mandell E.K., Otero J.H., Lundblad V. (2007). RPA-like proteins mediate yeast telomere function. Nat Struct Mol Biol.

[67] Miyake Y., Nakamura M., Nabetani A., Shimamura S., Tamura M., Yonehara S. (2009). RPA-like mammalian Ctc1-Stn1-Ten1 complex binds to single-stranded DNA and protects telomeres independently of the Pot1 pathway. Mol Cell.

[68] Wan B., Tang T., Upton H., Shuai J., Zhou Y., Li S. (2015 Dec). The Tetrahymena telomerase p75-p45-p19 subcomplex is a unique CST complex. Nat Struct Mol Biol..

[69] Bryan C., Rice C., Harkisheimer M., Schultz D.C., Skordalakes E. (2013). Structure of the human telomeric Stn1-Ten1 capping complex. PLoS One.

[70] Chandra A., Hughes T.R., Nugent C.I., Lundblad V. (2001). Cdc13 both positively and negatively regulates telomere replication. Genes Dev.

[71] Pennock E., Buckley K., Lundblad V. (2001). Cdc13 delivers separate complexes to the telomere for end protection and replication. Cell.

[72] Cohn M. (2013). OB fold contributes to telomere maintenance. Structure.

[73] Arcus V. (2002). OB-fold domains: a snapshot of the evolution of sequence, structure and function. Curr Opin Struct Biol.

[74] Flynn R.L., Zou L. (2010). Oligonucleotide/oligosaccharide-binding fold proteins: a growing family of genome guardians. Crit Rev Biochem Mol Biol.

[75] Evans S.K., Lundblad V. (1999). Est1 and Cdc13 as Comediators of Telomerase Access. Science.

[76] Mason M., Wanat J.J., Harper S., Schultz D.C., Speicher D.W., Johnson F.B. (2013). Cdc13 OB2 dimerization required for productive Stn1 binding and efficient telomere maintenance. Structure.

[77] Mitchell M.T., Smith J.S., Mason M., Harper S., Speicher D.W., Johnson F.B. (2010). Cdc13 N-terminal dimerization, DNA binding, and telomere length regulation. Mol Cell Biol.

[78] Qi H., Zakian V.A. (2000). The Saccharomyces telomere-binding protein Cdc13p interacts with both the catalytic subunit of DNA polymerase alpha and the telomerase-associated est1 protein. Genes Dev.

[79] Wu Y., Zakian V.A. (2011). The telomeric Cdc13 protein interacts directly with the telomerase subunit Est1 to bring it to telomeric DNA ends in vitro. Proc Natl Acad Sci U S A.

[80] Yu E.Y., Sun J., Lei M., Lue N.F. (2012). Analyses of Candida Cdc13 orthologues revealed a novel OB fold dimer arrangement, dimerization-assisted DNA binding, and substantial structural differences between Cdc13 and RPA70. Mol Cell Biol.

[81] Mason M., Skordalakes E. (2010). Insights into Cdc13 dependent telomere length regulation. Aging (Albany NY).

[82] Prescott J., Blackburn E.H. (1997). Functionally interacting telomerase RNAs in the yeast telomerase complex. Genes Dev.

[83] Yang C.P., Chen Y.B., Meng F.L., Zhou J.Q. (2006). *Saccharomyces cerevisiae* Est3p dimerizes in vitro and dimerization contributes to efficient telomere replication in vivo. Nucleic Acids Res.

[84] Sun J., Yang Y., Wan K., Mao N., Yu T.Y., Lin Y.C. (2011 Feb). Structural bases of dimerization of yeast telomere protein Cdc13 and its interaction with the catalytic subunit of DNA polymerase alpha. Cell Res..

[85] Derboven E., Ekker H., Kusenda B., Bulankova P., Riha K. (2014). Role of STN1 and DNA polymerase alpha in telomere stability and genome-wide replication in Arabidopsis. PLoS Genet.

[86] Grossi S., Puglisi A., Dmitriev P.V., Lopes M., Shore D. (2004). Pol12, the B subunit of DNA polymerase alpha, functions in both telomere capping and length regulation. Genes Dev.

[87] Huang C., Dai X., Chai W. (2012). Human Stn1 protects telomere integrity by promoting efficient lagging-strand synthesis at telomeres and mediating C-strand fill-in. Cell Res.

[88] Lue N.F., Chan J., Wright W.E., Hurwitz J. (2014). The CDC13-STN1-TEN1 complex stimulates Pol alpha activity by promoting RNA priming and primase-to-polymerase switch. Nat Commun.

[89] Petreaca R.C., Chiu H.C., Eckelhoefer H.A., Chuang C., Xu L., Nugent C.I. (2006). Chromosome end protection plasticity revealed by Stn1p and Ten1p bypass of Cdc13p. Nat Cell Biol.

[90] Li S., Makovets S., Matsuguchi T., Blethrow J.D., Shokat K.M., Blackburn E.H. (2009). Cdk1-dependent phosphorylation of Cdc13 coordinates telomere elongation during cell-cycle progression. Cell.

[91] Zhang W., Durocher D. (2010). De novo telomere formation is suppressed by the Mec1-dependent inhibition of Cdc13 accumulation at DNA breaks. Genes Dev.

[92] Liu C.C., Gopalakrishnan V., Poon L.F., Yan T., Li S. (2014). Cdk1 regulates the temporal recruitment of telomerase and Cdc13-Stn1-Ten1 complex for telomere replication. Mol Cell Biol.

[93] Tseng S.-F., Shen Z.-J., Tsai H.-J., Lin Y.-H., Teng S.-C. (2009). Rapid Cdc13 turnover and telomere length homeostasis are controlled by Cdk1-mediated phosphorylation of Cdc13. Nucleic Acids Res.

[94] Tseng S.F., Lin J.J., Teng S.C. (2006). The telomerase-recruitment domain of the telomere binding protein Cdc13 is regulated by Mec1p/Tel1p-dependent phosphorylation. Nucleic Acids Res.

[95] Wu Y., DiMaggio P.A., Perlman D.H., Zakian V.A., Garcia B.A. (2013). Novel phosphorylation sites in the *S. cerevisiae* Cdc13 protein reveal new targets for telomere length regulation. J Proteome Res.

[96] Mitton-Fry R.M., Anderson E.M., Theobald D.L., Glustrom L.W., Wuttke D.S. (2004). Structural basis for telomeric single-stranded DNA recognition by yeast Cdc13. J Mol Biol.

[97] Sun J., Yu E.Y., Yang Y., Confer L.A., Sun S.H., Wan K. (2009). Stn1-Ten1 is an Rpa2-Rpa3-like complex at telomeres. Genes Dev.

[98] Qian W., Wang J., Jin N.N., Fu X.H., Lin Y.C., Lin J.J. (2009). Ten1p promotes the telomeric DNA-binding activity of Cdc13p: implication for its function in telomere length regulation. Cell Res.

[99] Wellinger R.J. (2010). When the caps fall off: responses to telomere uncapping in yeast. FEBS Lett.

[100] DeZwaan D.C., Toogun O.A., Echtenkamp F.J., Freeman B.C. (2009). The Hsp82 molecular chaperone promotes a switch between unextendable and extendable telomere states. Nat Struct Mol Biol.

[101] Hang L.E., Liu X., Cheung I., Yang Y., Zhao X. (2011). SUMOylation regulates telomere length homeostasis by targeting Cdc13. Nat Struct Mol Biol.

[102] Puglisi A., Bianchi A., Lemmens L., Damay P., Shore D. (2008). Distinct roles for yeast Stn1 in telomere capping and telomerase inhibition. EMBO J.

[103] Nugent C.I., Hughes T.R., Lue N.F., Lundblad V. (1996). Cdc13p: a single-strand telomeric DNA-binding protein with a dual role in yeast telomere maintenance. Science.

[104] Webb C.J., Zakian V.A. (2012). Schizosaccharomyces pombe Ccq1 and TER1 bind the 14-3-3-like domain of Est1, which promotes and stabilizes telomerase-telomere association. Genes Dev.

[105] Shen Z.J., Hsu P.H., Su Y.T., Yang C.W., Kao L., Tseng S.F. (2014). PP2A and Aurora differentially modify Cdc13 to promote telomerase release from telomeres at G2/M phase. Nat Commun.

[106] Grandin N., Reed S., Charbonneau M. (1997). Stn1, a new Saccharomyces cerevisiae protein, is implicated in telomere size regulation in association with Cdc13. Genes Dev.

[107] Casteel D.E., Zhuang S., Zeng Y., Perrino F.W., Boss G.R., Goulian M. (2009). A DNA polymerase-{alpha}{middle dot}primase cofactor with homology to replication protein A-32 regulates DNA replication in mammalian cells. J Biol Chem.

[108] Gu P., Min J.N., Wang Y., Huang C., Peng T., Chai W., Chang S. (2012). CTC1 deletion results in defective telomere replication, leading to catastrophic telomere loss and stem cell exhaustion. EMBO J.

[109] Hsu C.L., Chen Y.S., Tsai S.Y., Tu P.J., Wang M.J., Lin J.J. (2004). Interaction of Saccharomyces Cdc13p with Pol1p, Imp4p, Sir4p and Zds2p is involved in telomere replication, telomere maintenance and cell growth control. Nucleic Acids Res.

[110] Nandakumar J., Bell C.F., Weidenfeld I., Zaug A.J., Leinwand L.A., Cech T.R. (2012). The TEL patch of telomere protein TPP1 mediates telomerase recruitment and processivity. Nature.

[111] Schmidt J.C., Dalby A.B., Cech T.R. (2014). Identification of human TERT elements necessary for telomerase recruitment to telomeres. Elife.

[112] Rao T., Lubin J.W., Armstrong G.S., Tucey T.M., Lundblad V., Wuttke D.S. (2014). Structure of Est3 reveals a bimodal surface with differential roles in telomere replication. Proc Natl Acad Sci U S A.

[113] Wang F., Stewart J.A., Kasbek C., Zhao Y., Wright W.E., Price C.M. (2012). Human CST has independent functions during telomere duplex replication and C-strand fill-in. Cell Rep.

[114] Calado R.T., Young N.S. (2009). Telomere diseases. N Engl J Med.

[115] Cawthon R.M., Smith K.R., O'Brien E., Sivatchenko A., Kerber R.A. (2003). Association between telomere length in blood and mortality in people aged 60 years or older. Lancet.

[116] Lamm N., Ordan E., Shponkin R., Richler C., Aker M., Tzfati Y. (2009). Diminished telomeric 3′ overhangs are associated with telomere dysfunction in Hoyeraal–Hreidarsson syndrome. PLoS One.

[117] Grandin N., Damon C., Charbonneau M. (2001). Ten1 functions in telomere end protection and length regulation in association with Stn1 and Cdc13. EMBO J.

